# Immunoprevention and immunomodulation of yellow fever: A scoping review of global and Latin American evidence

**DOI:** 10.1371/journal.pone.0352755

**Published:** 2026-07-14

**Authors:** Clímaco de Jesús Pérez Molina, Flor Elena Chavarro Bermeo, Arlin Martha Bibiana Pérez Hernández

**Affiliations:** 1 Master in Health Economics. Universidad El Bosque, Faculty of Medicine—Doctorate in Public Health Program, Bogota, Colombia; 2 Master in Physiology. Universidad El Bosque, Faculty of Medicine—Doctorate in Public Health Program, Bogota, Colombia; 3 Doctorate in Epidemiology and Public Health; Master in Public Health Program, Bogota, Colombia; Instituto Butantan, BRAZIL

## Abstract

**Background:**

Yellow fever (YF) remains a major global public health challenge, with recurrent outbreaks in Africa and the Americas despite the long-standing availability of a highly effective vaccine. In recent years, outbreaks in Brazil and Colombia (2024–2025) have underscored persistent barriers to achieving optimal vaccination coverage and have highlighted the predominantly reactive nature of many public health responses to YF transmission.

**Objective:**

To map and synthesize evidence published between 2020 and 2025 on YF immunoprevention and immunomodulation at global, Latin American, and Colombian levels, identifying advances, gaps, and implications for public health policy.

**Methods:**

A scoping review was conducted following PRISMA-ScR guidelines. Eligible sources included original research, reviews, and official documents addressing vaccination strategies, safety, and immune responses. Publications in English, Spanish, and Portuguese from January 2020 to September 2025 were retrieved from major databases and grey literature sources. Data were tabulated and descriptively synthesized.

**Results:**

Seventy-eight documents met inclusion criteria. The literature consistently supported the high efficacy of a single 17D vaccine dose, with fractional dosing validated as an emergency strategy in contexts of vaccine shortage. Immunological evidence confirmed strong and durable humoral and cellular responses, consistent with foundational studies conducted prior to 2020. However, important gaps persist, particularly regarding immunogenicity and safety in Latin American populations, older adults, pregnant women, and people living with HIV. In Colombia, universal vaccination campaigns during the 2025 outbreak improved coverage but revealed weaknesses in pharmacovigilance, local research capacity, and sustained prevention strategies.

**Conclusions:**

The 17D vaccine remains the cornerstone of YF control. However, sustaining elimination goals requires not only maintaining ≥95% vaccination coverage but also strengthening pharmacovigilance systems, generating locally relevant immunological evidence, and transitioning from reactive outbreak responses to more proactive, context-sensitive vaccination strategies.

## Introduction

Yellow fever (YF) is an acute hemorrhagic arboviral disease caused by a *Flavivirus* transmitted by mosquitoes of the genera *Haemagogus*, *Sabethes*, and *Aedes*, maintaining both sylvatic and urban transmission cycles with critical epidemiological implications [1]. Clinically, YF is characterized by sudden-onset fever, jaundice, hemorrhage, and multiorgan failure in severe forms, with case fatality rates reaching up to 60% in complicated cases [2]. Despite the availability of a highly effective vaccine for more than eight decades, YF continues to be regarded as a re-emerging disease and a persistent global health challenge due to ongoing transmission in endemic areas, suboptimal vaccination coverage in rural settings, and population movements that facilitate viral introduction into previously YF-free regions [3].

Globally, the World Health Organization (WHO) estimates 84,000–170,000 severe cases and up to 60,000 deaths annually, concentrated mainly in sub-Saharan Africa and the Amazon Basin [4]. Uncontrolled urbanization, deforestation, expansion of *Aedes aegypti* populations, and limited capacity of health systems to sustain optimal vaccination coverage have contributed to the reemergence of outbreaks in countries that had reported no autochthonous cases for decades [5]. Moreover, the impact of climate change has altered vector distribution patterns, expanding geographic risk areas [6].

In the Americas, YF has shown a notable resurgence in recent years. Between 2016 and 2019, Brazil reported over 2,000 confirmed human cases with high mortality, preceded by extensive epizootics in non-human primates [7]. Since 2020, cases have been reported in Peru, Bolivia, and more recently in Colombia, where the departments of Tolima and Putumayo documented outbreaks between 2024 and 2025 [8,9]. These recent events have renewed global interest in understanding the immunological mechanisms underlying YF control and the operational challenges of sustaining high vaccination coverage, particularly in contexts of intense population mobility and structural vulnerability.

Historically, Colombia has been affected by sylvatic YF and had maintained control through endemic-area vaccination strategies and entomological surveillance. However, the 2025 outbreak prompted a public health alert and intensified vaccination efforts [10]. The Ministry of Health and Social Protection issued External Circular 012, recommending universal vaccination of all individuals aged ≥9 months residing in high-risk municipalities, expanding the eligible age range and reinforcing pharmacovigilance systems [11].

However, beyond its immediate operational relevance, this policy decision also reflects a broader structural challenge in public health governance. It underscores the persistent tendency to implement interventions in a predominantly reactive manner, often triggered by the occurrence of outbreaks rather than guided by sustained, anticipatory prevention strategies. In the context of yellow fever—an infection for which a highly effective and safe vaccine has been available for more than eight decades—the need to expand vaccination coverage in response to emerging cases reveals underlying gaps in the continuity and territorial consolidation of immunization programs. Moreover, such reactive approaches may increase population vulnerability by allowing immunity gaps to persist until epidemiological pressure becomes evident, while simultaneously placing considerable strain on health system capacity during emergency responses. Therefore, these dynamics highlight the importance of transitioning toward proactive vaccination models, grounded in active epidemiological surveillance, systematic monitoring of coverage, and forward-looking risk assessment. From this perspective, maintaining sustained population immunity levels ≥95%—particularly in areas with ongoing sylvatic transmission or ecological suitability for vector expansion—should be considered a core public health priority rather than a response to crisis. In turn, the recent Colombian experience not only illustrates an effective outbreak response but also offers a critical opportunity to reframe vaccination governance toward more resilient, equitable, and prevention-oriented approaches. This policy shift highlights the importance of adapting national strategies to evolving epidemiological dynamics, consistent with Pan American Health Organization (PAHO) recommendations [12].

Immunoprevention through the live-attenuated yellow fever vaccine (17D) remains the primary strategy for YF control. Numerous studies confirm that a single dose provides lifelong protection in most immunocompetent individuals [13]. Nonetheless, debate persists over the need for booster doses in specific groups such as adults ≥60 years, individuals living with HIV, or those with immunocompromising conditions [14]. Both PAHO and WHO have endorsed the use of fractional doses (0.1 mL) as an exceptional measure during mass vaccination campaigns amid vaccine shortages, raising questions regarding the durability of immunity and correlates of protection under these circumstances [15].

From an immunomodulatory perspective, the yellow fever vaccine represents a paradigmatic model of durable antiviral immunity. It elicits neutralizing antibodies in more than 95% of vaccinees within two weeks of immunization [16] and induces robust activation of CD4+ and CD8 + T cells, generating long-term immunological memory [17]. Follow-up studies have demonstrated protective antibody titers persisting for decades after vaccination, supporting the single-dose policy [18]. However, important gaps remain in characterizing immune responses among special populations and in defining universally accepted immunological correlates of protection [19].

The resurgence of YF in Colombia and across the region highlights persistent knowledge and operational gaps: the lack of recent seroprevalence studies in local populations, limited pharmacovigilance to detect rare vaccine-associated events (such as yellow fever vaccine-associated viscerotropic disease [YEL-AVD] and yellow fever vaccine-associated neurotropic disease [YEL-AND]), and the absence of evidence on the immunogenicity of fractional-dose schedules implemented in Latin America. These limitations justify the need for a comprehensive synthesis of available literature to provide an integrated overview of current evidence and inform public health decision-making.

Accordingly, the objective of this scoping review was to map evidence published between 2020 and 2025 on YF immunoprevention and immunomodulation in global, Latin American, and Colombian contexts. Specifically, it aimed to identify advances and challenges regarding: **Population:** individuals at risk for or affected by YF, including special groups such as older adults, pregnant and lactating women, and people living with HIV; **Concept:** immunoprevention through yellow fever vaccination (including fractional and booster doses) and immunomodulation encompassing humoral and cellular immune responses; and **Context:** the global, regional (Latin American), and national (Colombian) levels of implementation and policy relevance.

## Methods

### Protocol and registration

This scoping review followed a predefined protocol developed in accordance with the PRISMA-ScR 2018 guidelines. It was developed Checklist for the Scoping Review (View supplementary information S1 Table) and submitted for registration in PROSPERO (International Prospective Register of Systematic Reviews; registration ID: CRD420251163262, October 7, 2025). The full protocol will be publicly available at https://www.crd.york.ac.uk/PROSPERO/view/CRD420251163262

### Study design

The review was conducted following the PRISMA-ScR (Preferred Reporting Items for Systematic Reviews and Meta-Analyses extension for Scoping Reviews) guidelines, aiming to map available evidence and identify knowledge gaps without restricting inclusion by methodological design [20]. This approach was selected given the expected heterogeneity of the sources (original research articles, reviews, technical reports, international guidelines, and national bulletins) and the need to capture both peer-reviewed and grey literature addressing YF immunoprevention and immunomodulation across diverse geographic contexts.

### Research question

The research question was formulated using the PCC framework (Population, Concept, Context) recommended for scoping reviews [21]:

- Population: individuals at risk for or affected by YF, including special populations (adults ≥60 years, pregnant and lactating women, people living with HIV, and immunocompromised individuals).
- Concept: immunoprevention (yellow fever vaccination, booster or fractional dosing, safety, and post-vaccination adverse events) and immunomodulation (humoral and cellular immune responses, immunological memory, and correlates of protection).
- Context: scientific and technical publications at the global, Latin American, and Colombian levels, published between January 1, 2020, and September 17, 2025.

The research question was aligned with the general objective described in the Introduction.

### Eligibility criteria

1. Original research articles, including observational studies, clinical trials, cohort studies, and seroprevalence surveys.
2. Narrative reviews, systematic reviews, meta-analyses, or prior scoping reviews.
3. Grey literature documents such as epidemiological alerts, technical reports, guidelines, and bulletins from recognized health authorities, included to complement peer-reviewed evidence with policy and surveillance data.
4. Accepted languages: English, Spanish, and Portuguese.
5. Publication period: January 2020 – September 2025.

Eligibility criteria were designed to capture the most recent and relevant evidence on YF immunoprevention and immunomodulation. The selected period (2020–2025) reflects scientific advances and public-health responses following major outbreaks in Brazil (2016–2019) and intensified vaccination campaigns in Latin America, particularly Colombia (2024–2025).

### Information sources

The following international databases were searched: PubMed/MEDLINE, Embase, Scopus, Web of Science, and the Cochrane Library. For regional literature in Spanish and Portuguese, LILACS, SciELO, and Redalyc were included. Grey literature was retrieved from official portals of WHO, PAHO, CDC, the Colombian National Institute of Health (INS), and the Ministry of Health and Social Protection. Preprint repositories (medRxiv, bioRxiv) were also screened, given their relevance for emerging evidence during outbreaks. All databases and grey-literature sources were searched from January 1, 2020, through September 17, 2025, to ensure inclusion of the most up-to-date publications and reports.

### Search strategy

A sensitive and reproducible search strategy was designed by combining controlled descriptors (MeSH/DeCS) and free-text terms using Boolean operators. Three main conceptual blocks and their synonyms were defined:

- Disease: “yellow fever,” *Yellow Fever* [MeSH], YF17D, 17D-204, 17DD.
- Intervention/Prevention: vaccine*, vaccination, immunization, immunoprevention, “fractional dose,” booster.
- Outcomes/Safety/Immunity: immunogenicity, “neutralizing antibodies,” “T cell,” “cellular immunity,” adverse event*, YEL-AVD, YEL-AND, immunomodulation.

Blocks were combined using AND, and synonyms within each block using OR. A publication-date filter (2020-01-01 to 2025-09-17) was applied. Quotation marks were used for exact phrases, truncation symbols (e.g., *vaccine*), and title/abstract fields were specified to improve precision when appropriate. The search strategy was iteratively refined to maximize relevant retrieval and minimize noise.

Example PubMed query: (“Yellow Fever”[Mesh] OR “yellow fever” OR “YF17D” OR “17D-204” OR “17DD”) AND (vaccine OR vaccination OR immunization OR immunoprevention OR “fractional dose” OR booster OR “adverse events” OR YEL-AVD OR YEL-AND OR immunogenicity OR “neutralizing antibodies” OR “T cell” OR “cellular immunity”) AND (“2020/01/01”[Date – Publication]: “2025/09/17”[Date – Publication])

For regional databases, contextual terms were added (e.g., *Latin America, South America, Colombia, Brazil, Peru, Bolivia*, and their equivalents in Spanish and Portuguese, such as *“fiebre amarilla,” “vacuna 17D,” “dosis fraccionada”*). Complete electronic search strings for all databases and grey-literature sources (PubMed, Scopus, Embase, LILACS, SciELO, and Web of Science) are presented in Table 1, in accordance with PRISMA-ScR transparency and reproducibility standards.

**Table 1:** Search strings in databases.

Database/ Source	Main string	Regional filter (optional)
PubMed/ MEDLINE	(“Yellow Fever”[Mesh] OR “yellow fever” OR “fiebre amarilla” OR “febre amarela” OR 17D OR “17D-204” OR 17DD) AND (vaccin* OR immuniz* OR “inmunización” OR “imunização” OR immunoprevention OR “inmunoprevención” OR “fractional dose” OR fraccionad* OR “dose fraction*” OR booster OR refuerzo OR “adverse event*” OR “YEL-AVD” OR “YEL-AND” OR immunogenic* OR “neutralizing antibod*” OR “anticuerpos neutralizantes” OR “cellular immun*” OR “T cell*” OR “linfocitos T”) AND (“2020/01/01”[Date – Publication]: “2025/12/31”[Date – Publication])	(Colombia OR Brazil OR Peru OR Bolivia OR “Latin America” OR “South America” OR “América Latina”)
Embase (Ovid)	(‘yellow fever’/exp OR ‘yellow fever’ OR ‘fiebre amarilla’ OR ‘febre amarela’ OR 17d OR ‘17d-204’ OR 17dd) AND (vaccin*:ti,ab,kw OR immuniz*:ti,ab,kw OR immunoprevention:ti,ab,kw OR ‘fractional dose’:ti,ab,kw OR fraccionad*:ti,ab,kw OR booster:ti,ab,kw OR ‘adverse event*’:ti,ab,kw OR ‘yel-avd’:ti,ab,kw OR ‘yel-and’:ti,ab,kw OR immunogenic*:ti,ab,kw OR ‘neutralizing antibod*’:ti,ab,kw OR ‘cellular immun*’:ti,ab,kw OR ‘t cell*’:ti,ab,kw) AND ([2020–2025]/py)	(colombia:ti,ab,kw OR brazil:ti,ab,kw OR peru:ti,ab,kw OR bolivia:ti,ab,kw OR ‘latin america’:ti,ab,kw OR ‘south america’:ti,ab,kw)
Scopus	TITLE-ABS-KEY (“yellow fever” OR “fiebre amarilla” OR “febre amarela” OR 17D OR “17D-204” OR 17DD) AND TITLE-ABS-KEY (vaccin* OR immuniz* OR immunoprevention OR “fractional dose” OR fraccionad* OR booster OR “adverse event*” OR “YEL-AVD” OR “YEL-AND” OR immunogenic* OR “neutralizing antibod*” OR “cellular immun*” OR “T cell*”) AND (PUBYEAR > 2019 AND PUBYEAR < 2026)	TITLE-ABS-KEY (Colombia OR Brazil OR Peru OR Bolivia OR “Latin America” OR “South America”)
Web of Science (Core Collection)	TS=(“yellow fever” OR “fiebre amarilla” OR “febre amarela” OR 17D OR “17D-204” OR 17DD) AND TS=(vaccin* OR immuniz* OR immunoprevention OR “fractional dose” OR fraccionad* OR booster OR “adverse event*” OR “YEL-AVD” OR “YEL-AND” OR immunogenic* OR “neutralizing antibod*” OR “cellular immun*” OR “T cell*”) Refined by: Publication Years=(2020–2025)	TS=(Colombia OR Brazil OR Peru OR Bolivia OR “Latin America” OR “South America”)
Cochrane Library	([mh “Yellow Fever”] OR “yellow fever” OR “fiebre amarilla” OR “febre amarela” OR 17D OR “17D-204” OR 17DD) AND (vaccin* OR immuniz* OR immunoprevention OR “fractional dose” OR fraccionad* OR booster OR “adverse event*” OR “YEL-AVD” OR “YEL-AND” OR immunogenic* OR “neutralizing antibod*” OR “cellular immun*” OR “T cell*”) WITH Publication Year from 2020 to 2025	—
Google Scholar	“yellow fever” OR “fiebre amarilla” OR “febre amarela” OR 17D OR 17DD AND (vaccin* OR immuniz* OR “fractional dose” OR fraccionad* OR booster OR “adverse events” OR YEL-AVD OR YEL-AND OR immunogenic* OR “neutralizing antibodies” OR “cellular immunity” OR “T cells”)	Colombia OR Brazil OR Peru OR Bolivia OR ‘Latin America’
EBSCOhost (Academic Search/ MEDLINE via EBSCO)	TX (“yellow fever” OR “fiebre amarilla” OR “febre amarela” OR 17D OR “17D-204” OR 17DD) AND TX (vaccin* OR immuniz* OR immunoprevention OR “fractional dose” OR fraccionad* OR booster OR “adverse event*” OR YEL-AVD OR YEL-AND OR immunogenic* OR “neutralizing antibod*” OR “cellular immun*” OR “t cell*”)	TX (Colombia OR Brazil OR Peru OR Bolivia OR “Latin America” OR “South America”)
Ovid (MEDLINE)	exp Yellow Fever/ OR (yellow fever OR fiebre amarilla OR febre amarela OR 17d OR 17dd OR 17d-204).ti,ab. AND (vaccin$ OR immuniz$ OR immunoprevention OR fractional adj3 dose OR fraccionad$ OR booster OR adverse adj2 event$ OR yel-avd OR yel-and OR immunogenic$ OR neutralizing adj2 antibod$ OR cellular adj2 immun$ OR t cell$).ti,ab. AND limit to yr = “2020 - 2025”	—
LILACS (BVS)	(“fiebre amarilla” OR “yellow fever” OR “febre amarela” OR 17D OR 17DD) AND (vacun* OR inmuniz* OR “dosis fraccionada” OR “fractional dose” OR refuerzo OR “eventos adversos” OR YEL-AVD OR YEL-AND OR inmunogenic* OR “anticuerpos neutralizantes” OR “inmunidad celular” OR “linfocitos T”)	(Colombia OR Brasil OR Perú OR Bolivia OR “América Latina”)
SciELO	(“fiebre amarilla” OR “yellow fever” OR “febre amarela” OR 17D OR 17DD) AND (vacun* OR inmuniz* OR “dosis fraccionada” OR “fractional dose” OR refuerzo OR “eventos adversos” OR YEL-AVD OR YEL-AND OR inmunogenic* OR “anticuerpos neutralizantes” OR “inmunidad celular” OR “linfocitos T”)	—
Redalyc	(“fiebre amarilla” OR “yellow fever” OR “febre amarela” OR 17D) AND (vacun* OR inmuniz* OR “dosis fraccionada” OR refuerzo OR “eventos adversos” OR inmunogenic* OR “inmunidad celular”)	—
Cochrane CENTRAL (Trials)	(yellow fever OR “fiebre amarilla” OR “febre amarela” OR 17D OR 17DD) AND (vaccin* OR immuniz* OR “fractional dose” OR fraccionad* OR booster OR immunogenic*) Publication Year: 2020–2025	—
medRxiv/ bioRxiv (preprints)	(“yellow fever” OR “fiebre amarilla” OR 17D OR 17DD) AND (vaccin* OR immuniz* OR “fractional dose” OR fraccionad* OR immunogenic* OR “neutralizing antibod*” OR “cellular immun*”) Date range: 2020–2025	—
PAHO/ WHO/ INS/ Colombian Ministry of Health (gray literature)	(“fiebre amarilla” OR “yellow fever”) AND (vacun* OR immuniz* OR “fractional dose” OR fraccionad* OR “pharmacovigilance” OR farmacovigilancia OR immunogenic*)	Colombia, circular, guideline, alert, bulletin, 2025

*Source: Authors’ elaboration*

### Selection of evidence sources

Study selection was conducted independently by two reviewers (authors of this study) following a two-stage screening process (title/abstract screening and full-text review). All records were initially screened based on titles and abstracts to assess potential eligibility according to the predefined inclusion criteria. Subsequently, full-text articles were independently evaluated by both reviewers to confirm eligibility.

Any discrepancies between reviewers during the screening and selection process were resolved through discussion and consensus. When consensus could not be reached, a third reviewer (author) was consulted to ensure methodological rigor and minimize selection bias.

**Fig 1:**
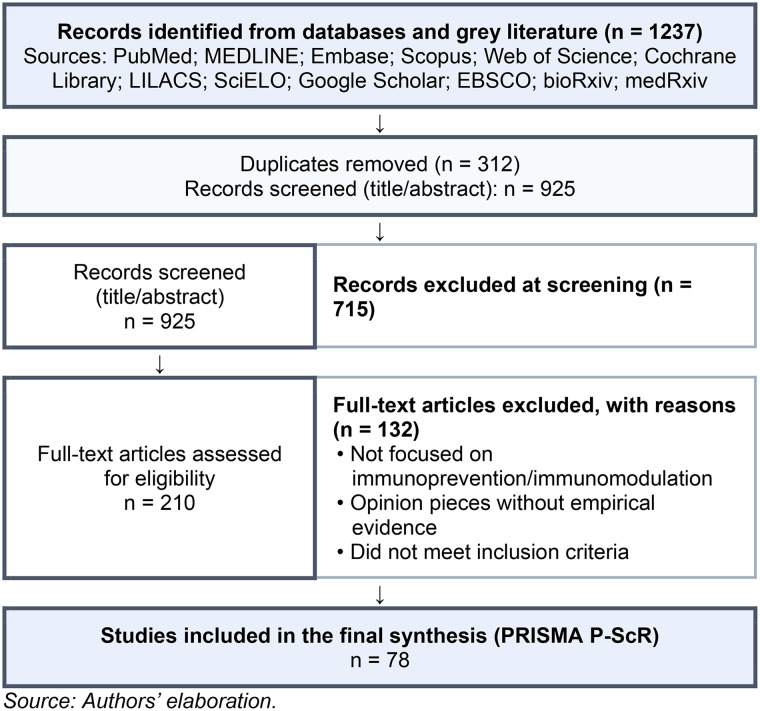
PRISMA-ScR flow diagram for the Yellow Fever Scoping Review (2020–2025).

The selection process and corresponding record counts at each stage are illustrated in Fig 1, developed following PRISMA-ScR flow-diagram standards to ensure methodological transparency.

### Data extraction

A standardized Microsoft Excel template was created to extract the following variables: author(s), year of publication, country or region, study design, population or target group, immunological or vaccination focus (immunoprevention or immunomodulation), reported outcomes (immunogenicity, safety, coverage, adverse events, or immune correlates), and key policy or programmatic implications. Data tabulation followed the methodological framework of Arksey and O’Malley, refined by Levac et al., and the Joanna Briggs Institute (JBI) guidance for scoping reviews, consistent with PRISMA-ScR.

### Data items

For each included document, the variables listed above were extracted. When multiple outcomes were reported, those most relevant to immunological or preventive aspects were prioritized. Incomplete data were recorded as “Not reported.” For this review, *immunoprevention* was defined as all interventions related to vaccination strategies and coverage—including fractional or booster dosing—whereas *immunomodulation* encompassed studies reporting immune (humoral, cellular, or molecular) responses and correlates of protection following 17D vaccination.

To ensure clarity and methodological transparency, included sources were categorized according to type of evidence, including original research articles, review articles, and grey literature (e.g., technical reports, guidelines, and epidemiological bulletins), in accordance with the PRISMA-ScR framework. This classification enabled a structured comparison of findings across heterogeneous sources.

Data extraction was adapted to the nature of each type of evidence. For original studies, variables such as study design, population characteristics, and immunological or vaccination outcomes were prioritized. For review articles, key synthesized findings and thematic conclusions were extracted. For grey literature, policy-related information, implementation strategies, and surveillance data were emphasized.

Extracted variables were subsequently used to inform the thematic synthesis by grouping findings into the predefined analytical domains of immunoprevention and immunomodulation. This method allowed for the identification of patterns, consistencies, and evidence gaps across different types of sources and geographical contexts, in line with established scoping review standards.

### Critical appraisal of individual sources

Given the exploratory nature of this scoping review, no formal critical appraisal or risk-of-bias assessment was conducted, following the methodological framework of Arksey and O’Malley and JBI guidance for scoping reviews. Nonetheless, all included documents were examined to ensure adherence to minimum eligibility criteria, credibility of the source, and methodological coherence. This decision aligns with the exploratory intent of scoping reviews, which aim to map available evidence rather than exclude studies based on methodological quality.

### Ethical considerations

As this study involved secondary analysis of published literature, ethical approval was not required. Nevertheless, the review adhered to principles of scientific transparency and reproducibility by explicitly documenting the search strategies and selection criteria.

### Synthesis of results

Data synthesis was descriptive and thematic, following JBI guidance for scoping reviews. Extracted data were grouped into two main analytical domains—immunoprevention and immunomodulation—and subsequently classified by geographic scope (global, Latin America, Colombia). Thematic mapping identified trends, evidence gaps, and policy implications. Frequency counts were applied to describe the distribution of study designs, populations, and immunological outcomes. Findings were summarized in tables and figures to facilitate interpretation and highlight consistencies and divergences across sources. No quantitative meta-analysis was performed due to heterogeneity in study designs and outcomes; synthesis was therefore descriptive and narrative. Summary tables of key study characteristics and PRISMA-ScR flow diagrams were developed to depict the selection process and ensure full transparency.

## Results

### Selection of evidence sources

A total of 1,237 records were identified through database and grey literature searches conducted up to September 17, 2025. After removing 312 duplicates, 925 unique records were screened based on titles and abstracts. Of these, 210 full-text articles were assessed for eligibility, and 78 documents met the inclusion criteria and were included in the final synthesis. The main reasons for exclusion were lack of relevance to the review question (n = 132), including studies not focused on immunoprevention or immunomodulation, opinion pieces without empirical evidence, and studies that did not meet the predefined inclusion criteria. The full selection process is presented in Fig 1, following PRISMA-ScR flow diagram standards to ensure transparency and reproducibility.

### Characteristics of included sources

Among the 78 included documents, 36 (46%) were original research articles, 22 (28%) were narrative or systematic reviews, and 20 (26%) corresponded to grey literature from international organizations (WHO, PAHO, CDC) and national institutions (Colombian Ministry of Health, INS). Geographically, 4 studies addressed the global context, 30 the Latin American regional context, and 20 the Colombian national context. This distribution is summarized in Table 2, under the subsection “Distribution of included sources.” Immunological dimensions and evidence gaps are detailed in Table 3, providing an integrated overview of methodological diversity, thematic focus, and contextual scope across the 78 sources.

**Table 2:** Distribution of Included Sources by Type and Geographical Scope (2020–2025).

Category	Subcategory	Number of Sources (n = 78)	Percentage (%)	Description/ Examples
Type of document	Original research articles	36	46.2	Observational studies, clinical trials, cohort studies, and seroprevalence surveys assessing vaccination efficacy, immunogenicity, or safety.
	Reviews (systematic or narrative)	22	28.2	Literature syntheses and meta-analyses on vaccination strategies, immune responses, or safety.
	Grey literature	20	25.6	Official documents from WHO, PAHO, CDC, and national institutions (Colombian Ministry of Health, INS), including technical guidelines, epidemiological bulletins, and outbreak reports.
Geographical scope	Global	28	35.9	Multicountry studies or WHO/CDC reports describing global YF control strategies, fractional dosing trials, and immunogenicity data.
	Latin America (regional)	30	38.5	Regional analyses or studies conducted in multiple Latin American countries, especially Brazil, Peru, Bolivia, and Colombia.
	Colombia (national)	20	25.6	National reports, institutional data, and outbreak evaluations focusing on YF vaccination and immunological responses within Colombia.
Focus area	Immunoprevention	44	56.4	Studies centered on vaccination coverage, safety, and fractional-dose implementation.
	Immunomodulation	34	43.6	Studies addressing humoral and cellular immune responses, correlates of protection, and immunological mechanisms post-17D vaccination.

*Source: Authors’ elaboration. Notes: Percentages are rounded to one decimal place. Sources include peer-reviewed publications and official institutional reports identified between January 2020 and September 2025. The distribution highlights the predominance of original research and the balanced representation of global, regional, and national evidence.*

**Table 3:** Evidence and gaps in immune components.

Immune component	Evidence	Gaps
Neutralizing antibodies	Rapid and durable induction (>95% within 10–14 days)	Limited data in Latin America
CD4 + /CD8 + T cells	Robust activation and long-term memory	Correlates not standardized
Immunological memory	Protection up to 40 years post-vaccination	Special populations under-studied

Source: *Authors’ elaboration*

### Findings on immunoprevention

#### Global context.

International literature reaffirmed the continued centrality of the yellow fever vaccine in YF control. Recent studies confirmed that a single dose provides lifelong protection in immunocompetent individuals, with >95% efficacy in preventing severe disease [1,13]. WHO and CDC maintain that routine boosters are unnecessary except for specific populations, including older adults, immunocompromised individuals, and those living with HIV [8].

A recurrent topic was the use of fractional doses (0.1 mL, equivalent to one-fifth of the standard dose). Clinical trials and follow-up studies demonstrated that this strategy maintains protective neutralizing antibody titers for at least 12 months [15]. WHO has incorporated fractional-dose vaccination as an emergency response strategy during global vaccine shortages, particularly in Africa and Latin America.

Safety reports indicated that mild adverse events (e.g., fever, myalgia, headache) occurred in 10–20% of vaccine recipients, while serious events such as vaccine-associated viscerotropic disease (YEL-AVD) and neurotropic disease (YEL-AND) were rare, with an incidence of 0.3–0.8 cases per million doses administered [26]. These findings reaffirm the favorable benefit–risk profile of yellow fever vaccination (see Table 4).

**Table 4:** Dose, risk, and benefit of immunization.

Topic	Findings	Source
Single dose	Lifelong protection in immunocompetent individuals	WHO 2013; CDC 2025
Fractional dose	0.1 mL effective in outbreak settings; comparable short-term	Brazil 2018; PAHO 2025
Adverse events	YEL-AVD/AND rare, < 1 per million doses	CDC; WHO
Special populations	Limited evidence in ≥60 years, HIV-positive, pregnant women	PAHO; CDC

*Source: Authors’ elaboration*

### Regional context

Across Latin America, vaccination was consistently reported as the cornerstone of outbreak containment in areas where sylvatic transmission expanded into new territories [7,12,22–24]. In Brazil, the 2016–2019 outbreaks prompted strategic adaptations such as the implementation of fractional-dose campaigns, which became a model for other countries [11]. In Peru, vaccination coverage in Amazonian regions reached 85%, though gaps persisted in Indigenous and remote rural communities with limited access [25].

In Bolivia, national health authorities faced logistical challenges in maintaining consistent vaccine supply in border regions [26]. In all cases, PAHO issued epidemiological alerts recommending expansion of vaccination coverage to travelers and high-risk populations [12]. The safety profile of the vaccine was consistently favorable throughout the region, though isolated reports of serious adverse events in older adults reinforced the recommendation of individualized risk assessment in this group [14].

### National context

In Colombia, the National Institute of Health (INS) and the Ministry of Health documented the 2024–2025 outbreaks [27]. In response, the Ministry issued *External Circular 012 (2025)* mandating universal vaccination for all individuals aged ≥9 months residing in or traveling to risk zones [27]. This policy aimed to rapidly achieve coverage above 95%, consistent with international standards.

National reports indicated that despite adequate vaccine supply, effective coverage in some municipalities remained below 80% due to geographic barriers, misinformation, and local logistical limitations in program implementation [11]. These findings exposed systemic weaknesses in community outreach and pharmacovigilance capacity that hindered uniform protection in high-risk populations.

### Findings on Immunomodulation

#### Global context.

Immunological evidence demonstrated that the yellow fever vaccine elicits a robust and durable immune response, characterized by rapid induction of neutralizing antibodies in over 95% of vaccinees within 10–14 days [28]. These antibodies have been shown to persist for decades in most individuals, supporting the single-dose policy [28]. Studies also confirmed activation of CD4+ and CD8 + T cells and establishment of long-term immunological memory, with the magnitude of the early cellular response correlating with the duration of protection [29].

### Regional context

Regional research on immunomodulation remains limited, with most studies originating from Brazil. Investigations assessing immune responses following fractional-dose campaigns revealed that short-term immunogenicity was comparable to that of full-dose vaccination, although antibody titers tended to decline more rapidly over time [30]. These findings highlight the need for long-term follow-up studies to determine the durability of protection.

Other Latin American studies examining immune responses in Indigenous and rural communities demonstrated variability in seroconversion rates influenced by nutritional factors and comorbidities [31,32]. This underscores the importance of context-specific immunological studies in populations facing social and health inequities.

### National context

In Colombia, available studies remain scarce and largely limited to institutional reports and historical seroprevalence surveys. During the 2025 outbreak, the INS announced the launch of a cohort study to evaluate the immunogenicity and safety of the 17D vaccine in adults aged ≥60 years; however, as of September 2025, no preliminary results had been published [33]. The absence of contemporary immunomodulatory data constitutes one of the most significant evidence gaps identified by this review.

### Summary of results

Across all evidence sources, findings on immunoprevention consistently confirmed the strong and sustained efficacy of the yellow fever vaccine, the safety and feasibility of fractional-dose administration, and its overall public health benefit for outbreak control. Nevertheless, the evidence base remains geographically uneven, with limited data from Latin America and Africa on implementation barriers, long-term surveillance, and vaccination coverage [13,18,24]. Regarding immunomodulation, most studies reported robust and long-lasting humoral and cellular responses following yellow fever vaccination, including durable neutralizing antibody titers and activation of T-cell memory. However, marked heterogeneity was observed in immune correlates and laboratory methodologies, and few studies explored innate immunity, systems biology perspectives, or transcriptomic profiles. The thematic synthesis revealed three major gaps: 1—lack of standardized immunological outcomes across studies; 2—limited evidence for special populations, including older adults, pregnant women, and individuals living with HIV; and 3—insufficient region-specific data from endemic countries, limiting generalizability and policy relevance. These gaps directly address the review’s objectives by mapping the global landscape of YF immunoprevention and immunomodulation, highlighting research priorities, and identifying policy implications for future action. Importantly, these findings are consistent with foundational studies conducted prior to 2020, which established the long-term immunogenicity, safety, and durability of protection conferred by the 17D vaccine, thereby providing the scientific basis upon which more recent evidence and public health strategies have been developed.

Regarding immunomodulation, most studies reported robust and long-lasting humoral and cellular responses following yellow fever vaccination, including durable neutralizing antibody titers and activation of T-cell memory. However, marked heterogeneity was observed in immune correlates and laboratory methodologies, and few studies explored innate immunity, systems biology perspectives, or transcriptomic profiles.

The thematic synthesis revealed three major gaps: 1-Lack of standardized immunological outcomes across studies. 2-Limited evidence for special populations, including older adults, pregnant women, and individuals living with HIV. 3-Insufficient region-specific data from endemic countries, limiting generalizability and policy relevance. These gaps directly address the review’s objectives by mapping the global landscape of YF immunoprevention and immunomodulation, highlighting research priorities, and identifying policy implications for future action.

## Discussion

The findings demonstrate the robust immune response induced by the yellow fever vaccine and identify persistent gaps in special populations and in regional implementation. This scoping review provides an integrated synthesis of global, regional, and local evidence published during the study period. The results consistently confirm the high efficacy and safety of the yellow fever vaccine, supporting the WHO (2013) recommendation—still widely upheld in current guidelines—of a single lifelong dose for immunocompetent individuals and the use of fractional doses during outbreaks as a feasible emergency strategy. Importantly, these conclusions are grounded in a substantial body of foundational research conducted prior to 2020, which established the immunological basis of long-term protection following 17D vaccination. Seminal studies by Stanley A. Plotkin and Eduardo Gotuzzo demonstrated that a single dose can induce protective immunity lasting for decades, supported by persistent neutralizing antibody titers and long-lived immunological memory. In parallel, mechanistic studies such as those by Rafael S. Akondy et al. (2015) provided deeper insight into the cellular immune response, showing robust activation of CD4+ and CD8 + T cells as key components of durable antiviral immunity. However, this recommendation—originally issued by WHO in 2013—has been the subject of ongoing scientific debate, particularly considering evidence generated in the subsequent decade. Between 2013 and 2020, several studies raised important questions regarding the durability of immunity in specific populations, including older adults, children vaccinated at early ages, and individuals with immunocompromising conditions. Longitudinal analyses have suggested that, although most immunocompetent individuals maintain protective neutralizing antibody titers for decades, a proportion may experience a gradual decline in immunity over time, potentially leading to susceptibility in the absence of booster doses. Moreover, research conducted in the context of fractional-dose vaccination strategies—implemented during vaccine shortages—has demonstrated adequate short-term immunogenicity but also pointed toward a more rapid waning of antibody levels compared with full-dose regimens. These findings have fueled a nuanced debate on whether a universal single-dose policy is sufficient across all populations and epidemiological contexts, or whether more tailored strategies, including booster doses for selected high-risk groups, should be considered. Therefore, while the current global consensus continues to support a single-dose approach for most individuals, the available evidence underscores the need for ongoing surveillance, long-term immunogenicity studies, and context-specific policy adaptations, particularly in endemic regions with heterogeneous risk profiles. However, geographical and contextual inequities persist—particularly in Latin America and Africa—where implementation and surveillance studies remain limited.

Evidence on immunomodulation highlights strong and long-lasting humoral and cellular immune responses, yet considerable heterogeneity remains in the definition of immune correlates, laboratory assays, and long-term follow-up. Few studies have addressed innate immune mechanisms, systems biology analyses, or vulnerable populations such as older adults, pregnant women, and people living with HIV. Overall, this synthesis aligns with the objectives of the review by mapping global and regional trends, identifying gaps in immunological standardization and population-based research, and emphasizing the relevance of these findings for global public health agencies such as WHO (2013), immunization programs, and translational research agendas focused on YF control [1–13]. The synthesis illustrates the coherence between immunological mechanisms and public health outcomes.

Furthermore, while the selected time frame (2020–2025) allowed for a focused synthesis of the most recent scientific advances and public health responses following major outbreaks in Latin America, this delimitation inevitably entails the exclusion of a substantial body of earlier research that has been foundational to the field. Decades of prior investigations have played a critical role in shaping current understanding of yellow fever immunobiology, vaccine safety, and the durability of protective immunity. Seminal studies conducted before 2020 established the long-term persistence of neutralizing antibodies, the role of cellular immune responses in sustained protection, and the overall favorable benefit–risk profile of the 17D vaccine—evidence that continues to underpin contemporary vaccination policies and WHO recommendations. Moreover, these earlier contributions were not exclusively driven by outbreak contexts, but rather emerged from sustained scientific efforts aimed at elucidating the fundamental mechanisms of antiviral immunity and vaccine performance. Therefore, while the present review prioritizes recent evidence to capture evolving epidemiological dynamics and policy responses, its findings should be interpreted in continuity with this broader historical knowledge base. Integrating insights from both recent and foundational studies is essential to avoid temporal bias and to ensure a more comprehensive understanding of yellow fever immunoprevention and immunomodulation across different epidemiological contexts.

In this broader context, when compared with international literature, findings from Latin America and Colombia reinforce the validity of this paradigm but also reveal tensions. In Brazil, for instance, the 2016–2019 outbreaks tested the health system and prompted the selective implementation of fractional-dose vaccination as an emergency strategy in regions facing vaccine shortages. Importantly, this approach was not applied uniformly across the country; in areas where routine yellow fever vaccination was already recommended and vaccine supply remained sufficient, the standard full-dose regimen continued to be used. This differentiated strategy reflects a context-specific adaptation of immunization policies, balancing the need to expand coverage rapidly in high-demand settings while preserving standard dosing practices where feasible. Martins et al. (2013) and Possas et al. (2018) demonstrated that this measure achieved immunogenicity comparable to the full dose during the first year, although questions remain regarding long-term durability. Subsequent studies, including those by Ana Carolina Campi-Azevedo et al. (2019), further refined this understanding by showing that, although short-term immune responses following fractional-dose vaccination are comparable to those induced by full doses, antibody titers may decline more rapidly over time, raising questions about the long-term durability of protection. In this regard, Brazilian studies provide valuable evidence that has not yet been replicated in other countries of the region.

In the Colombian context, the 2025 situation reflects the country’s epidemiological vulnerability. The outbreak in departments such as Tolima and Putumayo led to universal vaccination of all individuals aged ≥9 months, consistent with PAHO recommendations [33,34]. However, difficulties in achieving effective coverage above 95% exposed a gap between policy and practice—a situation also documented in Peru’s *Epidemiological Bulletin* (2024) and Bolivia’s *Yellow Fever Outbreak Report* (2023). This underscores the need for differentiated strategies targeting rural and Indigenous populations, where geographic, cultural, and access barriers limit campaign effectiveness.

Regarding vaccine safety, results are consistent with global literature, indicating that severe adverse events following yellow fever vaccination are extremely rare. These findings are supported by foundational large-scale safety analyses conducted prior to 2020, including studies by Nancy P. Lindsey et al., which established that the incidence of serious adverse events such as yellow fever vaccine-associated viscerotropic disease (YEL-AVD) and neurotropic disease (YEL-AND) remains below one case per million doses administered. Subsequent research further refined this risk profile by identifying specific populations—particularly older adults—as having a relatively increased susceptibility to severe adverse events, although the absolute risk remains low. In this context, studies such as those by Rafferty et al. (2013) highlighted the importance of individualized risk–benefit assessment in populations aged ≥60 years, especially in endemic or outbreak settings. Despite this well-established safety profile, important challenges persist in the implementation of robust pharmacovigilance systems, particularly in low- and middle-income countries. While countries such as Brazil have strengthened adverse-event monitoring frameworks following recent outbreaks, Colombia continues to face structural limitations in detection, reporting, and active surveillance, leading to potential underreporting of rare but clinically significant events. This gap underscores the need to move from passive to active pharmacovigilance models, integrating real-time surveillance, cohort monitoring, and risk stratification approaches to ensure both vaccine safety and public confidence in immunization programs [11].

With respect to immunomodulation, this review confirms that the yellow fever vaccine is a unique model of long-term antiviral immunity. The findings illustrate the 17D strain as a model of balanced activation of innate and adaptive immunity, linking molecular mechanisms to population-level protection. Globally, studies such as Akondy et al. (2015) have shown not only sustained production of neutralizing antibodies but also the activation of CD4+ and CD8 + T cells that support long-term immunological memory. Reports such as Gotuzzo et al. (2013) describe persistent protection up to 40 years after vaccination, reinforcing the international position that routine boosters are unnecessary.

However, regional findings nuance this consensus. In Brazil, Campi-Azevedo et al. (2019) emphasized that fractional-dose immunogenicity yields satisfactory short-term results compared with full-dose vaccination but exhibits faster antibody decline. This observation reinforces concerns raised in earlier studies regarding the potential need for booster strategies in specific epidemiological and population contexts. This raises critical questions: Will protection be equally durable? Could susceptible clusters emerge after a decade? Such uncertainties highlight the need for longitudinal studies across Latin America.

In Colombia, the absence of recent local immunogenicity studies represents a critical evidence gap. Although the INS announced in its 2025 document *Cohort Project on 17D Vaccine Immunogenicity in Older Adults* a study designed to evaluate immune responses in individuals aged ≥60 years, no results are yet available. This limits the country’s ability to base decisions on locally generated evidence, forcing extrapolation from other settings. The comparison with Brazil illustrates how the generation of local evidence strengthens health responses and guides adaptive policy.

Based on the results, several research gaps consistent with previous literature were identified. Together, these findings directly address the review’s objectives by demonstrating the robust immune response induced by the yellow fever vaccine and identifying persistent gaps in regional implementation and vulnerable populations:1-Correlates of immune protection. Although neutralizing antibodies remain the most widely accepted marker, there is no universally standardized threshold for predicting long-term immunity (Plotkin, 2010), limiting interpretation of strategies such as fractional dosing. 2-Special populations. According to Yactayo et al. (2020), evidence regarding older adults, pregnant and lactating women, and people living with HIV remains limited. Compared with Africa—where more studies have been conducted—Latin America shows an evident need for further research. 3-Pharmacovigilance. Underreporting of adverse events hinders accurate assessment of safety profiles. According to PAHO (2024), this issue is more pronounced in Colombia, contrasting with more consolidated systems in Brazil.4-Seroprevalence studies. As shown in the *National Seroprevalence Study in Colombia 2010–2012* (INS, 2013), the lack of updated population immunity data impedes accurate estimation of outbreak risk and campaign effectiveness.

In summary, while global consensus on the efficacy and safety of the yellow fever vaccine remains solid, the Latin American and Colombian contexts introduce important nuances related to access, surveillance, and the generation of locally relevant evidence. Positioning these findings within the broader body of pre-2020 research allows for a more comprehensive understanding of the evolution of scientific knowledge in this field, linking foundational immunological evidence with current epidemiological and programmatic challenges. This gap between global knowledge and local needs underscores the importance of strengthening regional research capacity and adapting public health policies to diverse epidemiological and social contexts. Furthermore, the critical analysis of reactive versus proactive vaccination approaches, together with the nuanced discussion on fractional dosing and long-term immunity, highlights the need for more anticipatory, context-sensitive immunization strategies. For Colombia, the implications are clear: (1) achieve homogeneous vaccination coverage ≥95% in endemic and high-risk areas; (2) strengthen pharmacovigilance systems to ensure timely detection of rare adverse events; (3) promote locally grounded immunogenicity and seroprevalence studies; and (4) ensure the long-term sustainability of vaccination programs under logistical and financial constraints. Experiences from countries such as Brazil and Peru demonstrate that programmatic resilience depends not only on vaccine availability but also on the capacity to build community trust, implement adaptive strategies, and maintain robust surveillance systems. Taken together, these considerations reinforce the manuscript’s contribution to both the scientific understanding of yellow fever immunoprevention and the development of more resilient, equitable, and evidence-informed public health strategies in endemic settings.

This review has several limitations inherent to scoping designs. The search was restricted to documents published in three languages, which may have led to the omission of relevant studies in other languages or outside the study period. The diversity of study designs and data types precluded a formal critical appraisal and quantitative meta-analysis. Partial reliance on grey literature may have introduced availability or reporting bias, particularly in countries with limited local research capacity. Furthermore, because the synthesis was primarily descriptive, the strength of conclusions depends on the methodological consistency and completeness of included sources. Despite these limitations, the review offers a broad and transparent overview of global and regional evidence on YF immunoprevention and immunomodulation, providing valuable insights for guiding future empirical research and policymaking.

## Conclusions

This scoping review achieved its objectives by mapping global, regional, and national evidence on YF immunoprevention and immunomodulation between 2020 and 2025. The evidence confirms that the live-attenuated yellow fever vaccine remains the cornerstone of YF control, providing durable protection after a single dose. Fractional-dose administration constitutes a viable strategy during supply shortages, though its long-term immunogenicity requires further evaluation.

The vaccine induces robust humoral and cellular immune responses; however, gaps persist in defining immune correlates of protection, evaluating vaccine responses in special populations, and strengthening local pharmacovigilance systems. To sustain elimination goals, countries must maintain coverage ≥95%, improve pharmacovigilance, and generate locally anchored immunological data to guide policy. For Latin America, addressing logistic and research inequities remains essential to ensure equitable and sustainable YF control.

## Supporting information

S1 Table PRISMA-ScR checklist. Checklist for the Preferred Reporting Items for Systematic Reviews and Meta-Analyses extension for Scoping Reviews (PRISMA-ScR), including all items addressed in this manuscript.
(DOCX)
