# Supplementary material for: Immunoprevention and immunomodulation of yellow fever: A scoping review of global and Latin American evidence
Source: PLoS One. 2026 Jul 14;21(7):e0352755. doi: 10.1371/journal.pone.0352755 (PMC13367723; doi:10.1371/journal.pone.0352755)
Supplement: S1 Table — Checklist for the Preferred Reporting Items for Systematic Reviews and Meta-Analyses extension for Scoping Reviews (PRISMA-ScR), including all items addressed in this manuscript. (DOCX) [file pone.0352755.s001.docx]

# PRISMA-ScR 2018 Checklist for the Scoping Review

Title: Immunoprevention and Immunomodulation of Yellow Fever: A Scoping Review of Global and Latin American Evidence

Authors: Clímaco de Jesús Pérez Molina, Flor Elena Chavarro-Bermeo, Arlin Martha Bibiana Pérez Hernández

Institution: Universidad El Bosque, Faculty of Medicine – Doctorate in Public Health Program (Bogotá, Colombia)

| Section | Item No. | Checklist Item (Official wording) | Location in Manuscript (page/section) | Author Comment / Observation |
| --- | --- | --- | --- | --- |
| Title | 1 | Identify the report as a scoping review. | Title page, Abstract | Yes – Clearly identified as a scoping review in title and abstract. |
| Abstract | 2 | Provide a structured summary including background, objectives, eligibility criteria, sources, methods, results, and conclusions. | Abstract | Yes – Structured abstract with all required components. |
| Introduction | 3 | Describe the rationale for the review in the context of what is already known. | Introduction (pp. 2–5) | Yes – Contextualized within recent outbreaks and immunological gaps. |
| Introduction | 4 | Provide an explicit statement of the questions and objectives being addressed. | End of Introduction | Yes – PCC framework clearly defined. |
| Methods | 5 | Indicate whether a protocol exists and where it can be accessed (e.g., PROSPERO). | Methods – Protocol and Registration | Yes – Registered in PROSPERO (CRD42001163262, 2025). |
| Methods | 6 | Specify eligibility criteria for sources of evidence. | Methods – Eligibility Criteria | Yes – Defines inclusion/exclusion, language, and period (2020–2025). |
| Methods | 7 | Describe information sources and date last searched. | Methods – Information Sources | Yes – Lists databases and date (September 17, 2025). |
| Methods | 8 | Present the full electronic search strategy for at least one database, including limits used. | Methods – Search Strategy, Table 1 | Yes – Complete PubMed strategy included. |
| Methods | 9 | State the process for selecting sources of evidence (screening, eligibility, inclusion). | Methods – Selection of Evidence Sources | Yes – PRISMA-ScR flow diagram described (Figure 1). |
| Methods | 10 | Describe the data charting process (data extraction methods). | Methods – Data Extraction | Yes – Standardized Excel template described. |
| Methods | 11 | List and define all variables and data items sought. | Methods – Data Items | Yes – Includes immunogenicity, safety, coverage, immune correlates, etc. |
| Methods | 12 | If done, describe methods used for critical appraisal of individual sources. | Methods – Critical Appraisal | Yes – No formal appraisal; rationale explained per JBI guidance. |
| Methods | 13 | Describe methods for handling and summarizing charted data. | Methods – Synthesis of Results | Yes – Thematic and descriptive synthesis following JBI guidance. |
| Results | 14 | Provide numbers of sources screened, assessed for eligibility, and included, with reasons for exclusions. | Results – Selection of Evidence Sources | Yes – Figure 1 flow diagram with counts and exclusions. |
| Results | 15 | Present characteristics of sources of evidence for each included study or source. | Results – Characteristics of Included Sources, Table 2 | Yes – Summarized by design and geographic scope. |
| Results | 16 | Present results of individual sources of evidence as relevant to the review question. | Results – Findings on Immunoprevention and Immunomodulation | Yes – Key findings synthesized by context. |
| Results | 17 | Present a summary of the evidence (e.g., main concepts, themes, types of evidence). | Results – Summary of Results | Yes – Summarizes key immunological outcomes and evidence gaps. |
| Results | 18 | Describe any critical appraisal of included sources, if conducted. | Methods – Critical Appraisal | NA – Not applicable; scoping review design per Arksey & O’Malley. |
| Discussion | 19 | Summarize main results (including overview of concepts, themes, types of evidence, and gaps). | Discussion (pp. 30–37) | Yes – Discusses strengths, limitations, and evidence gaps. |
| Discussion | 20 | Discuss limitations of the scoping review process. | Discussion – Limitations paragraph | Yes – Addresses language, grey literature, and heterogeneity limitations. |
| Conclusions | 21 | Provide a general interpretation of results with implications for research, policy, or practice. | Conclusions (final section) | Yes – Highlights implications for vaccination policy and immunological research. |
| Funding | 22 | Describe sources of funding for the review and role of the funders. | Acknowledgments / Funding | Yes – Declares no specific funding; institutional affiliation listed. |

Declaration: This checklist was completed based on the submitted version of the manuscript and confirms adherence to PRISMA-ScR 2018 items.

Signed:
Clímaco de Jesús Pérez Molina, PhD – Corresponding Author
Bogotá, Colombia – October 2025
